# IL-17 and IL-11 GCF Levels in Aggressive and Chronic Periodontitis Patients: Relation to PCR Bacterial Detection

**DOI:** 10.1155/2012/174764

**Published:** 2012-11-26

**Authors:** Olfat G. Shaker, Noha A. Ghallab

**Affiliations:** ^1^Department of Medical Biochemistry & Molecular Biology, Faculty of Medicine, Cairo University, Egypt; ^2^Department of Oral Medicine, Periodontology and Diagnosis, Faculty of Oral and Dental Medicine, Cairo University, Egypt

## Abstract

*Objectives*. This study evaluated IL-17 and IL-11 in gingival crevicular fluid (GCF) of generalized chronic periodontitis (GCP) and generalized aggressive periodontitis (GAgP) patients in relation to periodontopathic bacteria. *Subjects and Methods*. GCF samples were collected from 65 subjects including 25 CP, 25 GAgP, and 15 controls (C) and analyzed for IL-17 and IL-11 by an enzyme-linked immunosorbent assay. Molecular detection of bacteria in the dental plaque was determined by polymerase chain reaction. *Results*. The total amount of IL-17 was significantly higher in GAgP group than in GCP and C groups (*P* < 0.001). The IL-11 concentration was significantly higher in C and GCP groups than GAgP group (*P* < 0.001). The IL-11/IL-17 ratio was significantly higher in the C group than in GCP and GAgP groups (*P* < 0.05). Moreover, GAgP group showed lower ratios of IL-11/IL-17 when compared to GCP group. The high positivity of *P. gingivalis* in the dental plaque was associated with significantly increased GCF levels of IL-17 in GCP and GAgP patients. *Conclusions*. The increased IL-17 level in GCF of GAgP suggests a potential role in the aetiopathogenesis. Meanwhile, the decreased ratio of IL-11/IL-17 might reflect an imbalance between the proinflammatory and anti-inflammatory cytokines in different periodontal diseases.

## 1. Introduction

Periodontitis is a chronic inflammatory disease characterized by destruction of tooth-supporting tissues [1]. The red-complex bacteria including, *Porphyromonas gingivalis*, *Tannerella forsythia* and *Treponema denticola* were shown to have the closest association with the severity of periodontitis [2, 3] besides this group, *Aggregatibacter actinomycetemcomitans* and *Prevotella intermedia.* A complex cytokine network is synthesized in response to these periodontopathogenic bacteria [4] and plays an important role in periodontal disease [5]. The structure of bioactive bacterial compounds differs substantially between different bacteria and subsequently resulting in differences in cytokine production [6]. 

Exciting new evidence has emerged introducing a novel subset of T-helper (Th) lymphocytes termed “Th17” [7]. The role of Th17 cells and their specific cytokines (IL-17) in periodontal disease is just beginning to be investigated [8]. IL-17 is a proinflammatory cytokine that stimulates a variety of cells to produce inflammatory mediators such as IL-1, IL-6, and TNF-*α* [9]. Although Th17 pathways are mostly associated with protection against bacteria via recruitment of phagocytes [10], they have also been proposed to enhance alveolar bone resorption [11]. Previous studies showed that IL-17 was associated with chronic periodontitis [11–13] suggesting that it may contribute to periodontal tissue destruction.

IL-11 as an anti-inflammatory mediator has been shown to play an important role in the modulation of immune response via reduction of proinflammatory cytokine production in animal models [14]. It was predicted that IL-11 may be considered as a candidate molecule for therapeutic modulation of the host response in the management of periodontal diseases [5, 15, 16].

Regardless of the different clinical profiles between generalized aggressive periodontitis (GAgP) and generalized chronic periodontitis (GCP), it is not clear whether those patients present a distinct profile of inflammatory mediators [17]. Based on the above, the aim of this study was to determine the total amount and concentration of the cytokines IL-17 and IL-11 in the gingival crevicular fluid (GCF) from GCP, GAgP patients, and healthy controls (C) and to investigate these levels in relation to the periodontal pathogens presented in their dental plaque. To date this is the first study to examine the GCF IL-17 and IL-11 levels in GAgP and GCP patients in connection with periodontopathic bacteria in the dental plaque.

## 2. Materials and Methods

### 2.1. Study Population

Sixty-five systemically healthy subjects were selected from the Outpatient Clinic, Department of Oral Medicine and Periodontology, Faculty of Oral and Dental Medicine, Cairo University, between February 2010 and December 2010, who signed an informed consent form approved by the University Institutional Review Board after explaining the study. Exclusion criteria were as follows: pregnant women, subjects with <22 permanent teeth, having any given systemic disease, taking any type of medication and/or antibiotic therapy during the 3 months before the study, receiving previous periodontal treatment, and former or current smokers.

### 2.2. Studied Groups

Subjects with GAgP and GCP and healthy control (C) subjects were diagnosed based on the periodontal classification of The American Academy of Periodontology [1] and met the following criteria [18].

- GCP group (*n* = 25): GCP patients were >35 years of age and had a minimum of six teeth with at least one site each with pocket depth (PD) and clinical attachment level (CAL) >5 mm.
- GAgP group (*n* = 25): GAgP patients were <35 years of age and had a minimum of six teeth other than the first molars and incisors with at least one site each with PD and CAL >5 mm, and familial aggregation (subjects were asked if they had at least one other member of the family presenting or with a history of periodontal diseases).
- Control group (*n* = 15): C subjects were >20 years of age and had clinically healthy gingiva with zero plaque index, gingival index, and CAL ≤3 mm PD.

### 2.3. Clinical Parameters

All subjects received clinical examination including the following periodontal parameters: plaque index (PI), gingival index (GI), PD and CAL. One examiner performed all the measurements at six sites for all teeth mesiobuccal, mesiolingual, midbuccal, distobuccal, distolingual, and midlingual. Calibration exercises for probing measurements were performed in five patients before the actual study. The intraexaminer agreement was good, with a 0.84 *κ* value. PI was established by measuring the presence or absence of supragingival biofilm with a sweeping movement of the probe around the buccal, mesial, distal, and lingual regions of all teeth [19]. Marginal gingival bleeding was recorded with GI. PD was measured from the free-gingival margin to the base of the periodontal pocket and CAL was measured from the cementoenamel junction of the tooth to the base of the periodontal pocket. Measurements were rounded to the highest whole millimeter using the Michigan 0 probe with Williams' markings.

### 2.4. Dental Plaque and GCF Samples Collection

Following the careful removal of supragingival biofilm, areas were washed with water spray, isolated with cotton rolls, and gently dried. Subgingival biofilm samples were collected by sterile endodontic paper points (no. 35), which were inserted in the site with deepest periodontal pocket in each quarter and kept there for 30 s. The paper points were placed into sterile tubes containing 300 *μ*L PBS [20]. GCF was collected from the same sites of microbiological sampling. After subgingival biofilm collection, teeth were washed again, and the area was isolated and gently dried. GCF was collected by placing filter paper strips (Perio-paper, IDE Interstate, Amityville, NY, USA) into the pocket until a slight resistance was perceived and then left in place for 30 s. Strips contaminated by blood were excluded. Immediately, the volume of the sample was measured with the aid of a calibrated Periotron 800 (Oraflow Inc., Amityville, NY, USA). After volume measurements, the strips were placed into sterile eppendorf tubes containing 300 *μ*L PBS. All samples (subgingival biofilm and GCF) were immediately stored at −20°C until subsequent analysis. Only one examiner, the same one charged with clinical measures, collected all microbial and GCF samples.

### 2.5. Determination of IL-17 and IL-11 in GCF Samples

GCF samples were analyzed for IL-17 and IL-11 using commercially available human enzyme-linked immunosorbent assay (ELISA) kits (BioSource Europe SA, Nivelles, Belgium, and PromoCell, Heidelberg, Germany, resp.). Analyses were performed according to the manufacturer's protocol. All ELISA determinations were performed in duplicate. It is a sandwich-type ELISA where a monoclonal anti-human IL-17 and IL-11, adsorbed onto microwells, bind IL-17 and IL-11 in the sample, respectively. Results were calculated using the standard curves included in each assay kit. The intensity of the color was measured at 450 nm. The total amount of IL-17 and IL-11 was determined in picograms (pg) and calculations of the concentration in each sample were performed by dividing the total amounts of IL-17 by the volume of the sample (pg/*μ*L).

### 2.6. PCR for Dental Plaque Bacterial Detection

DNA was extracted from microbial samples using the DNA extraction kit (Roche, Mannheim, Germany) according to the manufacturer's recommendations. The conventional polymerase chain reaction (PCR) amplification of the conserved region of 16S ribosomal DNA was tested for periodontal pathogens including *A. actinomycetemcomitans*, *P. intermedia, P. gingivalis*, *T. forsythia*, and *T. denticola *(Table 1). All these PCR primers were obtained commercially (Gibco BRL, São Paulo, SP, Brazil). 100 ng of genomic DNA was added to the PCR mixture which contained 1 *μ*mol/L of the primers, 2.5 U of *Taq *polymerase in 1x buffer, and 0.2 mMol/L of dCTP, dGTP, dATP, and dTTP in a total volume of 50 *μ*L. PCR amplification was performed for 35 cycles of 30 s at 95°C, 30 s at 55°C, and 60 s at 72°C in thermocycler. Twenty *μ*L of each PCR reaction mixture was electrophoresed in 1.7% agarose gel in TAE buffer, and the amplification products were visualized under ultraviolet light, on ethidium bromide-stained gel.

### 2.7. Statistical Analysis

Numerical data were presented as mean and standard deviation (SD) values. Qualitative data were presented as frequencies and percentages. One-way ANOVA (Analysis of Variance) was used to compare mean PD and CAL in the three groups. Tukey's test for pair-wise comparisons was used to determine significant differences between groups when ANOVA test was significant. Kruskall-Wallis test was used to compare cytokine levels, PI, and GI in the three groups. Mann-Whitney *U* test was used in the procedure of pair-wise comparisons when Kruskall-Wallis test was significant and was also used to compare cytokine levels in positive and negative bacteriological cases. Chi-square (*x* ^2^) test was used to compare prevalence of different bacterial species in the three groups. The significance level was set at *P* ≤ 0.05. Statistical analysis was performed with SPSS 18.0, Chicago, IL, USA.

## 3. Results

### 3.1. The Clinical Characteristics of the Patients

The periodontal values of the studied groups were shown in Table 2. All of the clinical parameters were significantly higher in GAgP and GCP groups compared to C group. PI, GI, and CAL values were significantly higher in GAgP group than GCP group. 

### 3.2. Total Amount and Concentrations of IL-17 and IL-11 in GCF

The GCF volume, total amounts, and concentrations of IL-17 and IL-11 are shown in Table 3. The GCF volume was found to be the highest in GAgP group (significantly higher than GCP and C groups, *P* < 0.001), followed by GCP and C groups, respectively.

The total amount of IL-17 was significantly higher in GAgP group than in GCP and C groups (*P* < 0.001). The total amount of IL-17 was higher in GCP group than in C group but without statistical significance. Meanwhile, GAgP group had significantly lower IL-17 concentrations than GCP and C groups (*P* < 0.001). The difference between the GCP and C groups was also found to be significantly (*P* < 0.001) higher in C group (*P* < 0.001). The concentration of IL-11 was significantly higher in C and GCP groups than in GAgP group (*P* < 0.001). The GAgP group had significantly lower IL-11 concentrations when compared to GCP group (*P* < 0.001). No significant differences were found among the groups in the total amount of IL-11 (*P* = 0.309).

### 3.3. Cytokine Ratios

The IL-11/IL-17 ratios, total amount, and concentration are shown in Table 4. The IL-11/IL-17 ratio was significantly higher in the C group than GAgP and GCP groups (*P* < 0.05). GAgP group showed lower ratios of IL-11/IL-17 when compared to GCP group; yet no statistically significant difference was observed between them.

### 3.4. Bacterial Detection by PCR

The occurrence of different periodontal bacteria, *A. actinomycetemcomitans, T. forsythia, T. denticola, P. gingivalis*,* and P. intermedia*, in the dental plaque of GCP and GAgP patients is given in Table 5. Detection frequency and percentage of *A. actinomycetemcomitans *were significantly higher in GAgP than in the GCP patients. Meanwhile, the detection frequency and percentage of *T. denticola*, *P. gingivalis*, and *P. intermedia* were significantly higher in GCP than in the GAgP patients. No statistically significant difference was detected in the frequency of *T. forsythia* between GAgP and GCP patients yet it was higher in GCP patients.

### 3.5. Relationship between Bacterial Status and GCF Cytokine Level

**Figure 1:**
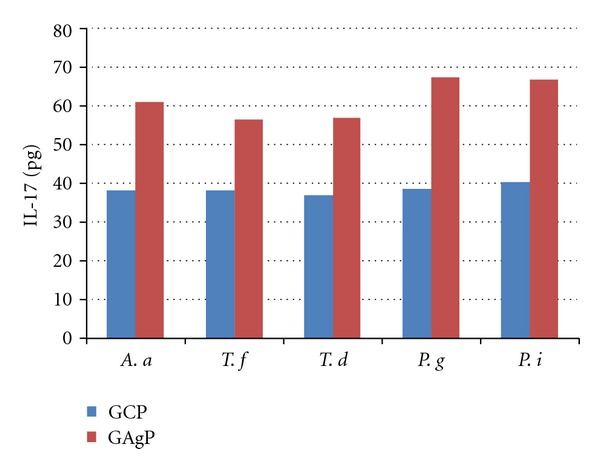
Mean GCF levels of total IL-17 based on the presence of different periodontal bacteria in the dental plaques of patients with GCP and GAgP.

**Figure 2:**
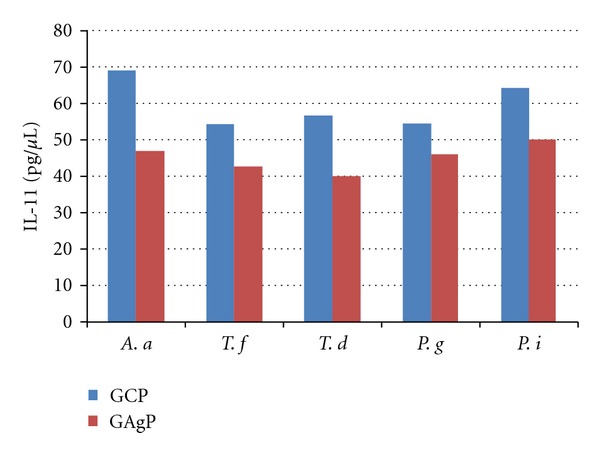
Mean GCF levels of IL-11 concentration based on the presence of different periodontal bacteria in the dental plaques of patients with GCP and GAgP.

Figures 1 and 2 show the relationship between GCF levels of IL-17 and IL-11 and the occurrence of different bacteria (*A. actinomycetemcomitans, T. forsythia, T. denticola, P. gingivalis*,* and P. intermedia*) in the positive samples of GCP and GAgP patients. The high occurrence of *P. gingivalis *in the dental plaque was associated with significantly increased GCF levels of IL-17 in GCP (*P* < 0.05) and GAgP patients (*P* < 0.001) in positive samples compared to negative samples. No significant differences in the GCF levels of the IL-11 depending on the presence of any periodontal bacteria were found (data not shown).

## 4. Discussion

The intensity, duration, and resolution of inflammation depend on shifting the balance between the activities of proinflammatory and anti-inflammatory cytokines during the periodontal inflammation [21]. Accordingly, this investigation evaluated the total amount and concentration of IL-17 and IL-11 in GCF of GCP, GAgP, and C subjects. 

The current study shows, for the first time, an overexpression in the total amount of IL-17 in GCF of GAgP patients compared to GCP and C ones. This might suggest a potential cellular hyperactivity that may favor periodontal destruction in GAgP. Recently, Duarte et al. [17] demonstrated that subjects with GAgP presented higher serum levels of IL-17 than subjects with GCP and C, and that this increase was related to gene polymorphisms coding for the synthesis of inflammatory mediators. Likewise, Schenkein et al. [22] detected IL-17 in sera from GAgP patients, suggesting that it could be from the locally produced cytokine in the periodontal tissues. GAgP patients are known to exhibit defective neutrophils chemotactic responses and enhanced oxidative metabolic responses [23]. It has been assumed that elevated IL-17 levels noted in GAgP may represent a compensatory increase in cytokine production in response to these functional defects [22]. Conversely, Borch et al. [24] found no differences between C and GAgP subjects in the release of IL-17 by mononuclear cells, yet results from cell-culture methodologies are not directly comparable with cytokines GCF levels.

This study also showed comparable results to Vernal et al. [12] who reported significantly higher total amounts of IL-17 in GCF and in the culture supernatants of GCP patients than in C. Elevated tissue concentrations of IL-17 could promote periodontitis progression by increasing concentrations of bone resorbing chemokines, which suggests that IL-17 may be a mediator of periodontal destruction [12, 25]. Previous studies showed that IL-17 induced the expression of prostaglandin E_2_ (PGE_2_) [26]. Others demonstrated that IL-17 has a synergy with TNF-*α* in synthesizing IL-6 and increasing bone resorption [27]. Interestingly, IL-17 has been observed to act on osteoblasts inducing their differentiation into mature osteoclasts [28, 29].

Alternatively, this study demonstrated a significant decrease in IL-17 GCF concentration in patients with GCP and GAgP compared with C, and in GAgP patients when compared with GCP patients. These results were supported by Vernal et al. [12] who reported lower IL-17 GCF concentration in GCP patients compared to C and attributed this decrease to the higher GCF volume produced in diseased sites with PD >5 mm than in healthy sites from C.

Johnson et al. [31] reported lower IL-11 concentrations within gingiva adjacent to deep PD, suggesting an imbalance between pro- and anti-inflammatory mediators in their study. This investigation also observed significantly lower IL-11 concentrations in GAgP group which had the deepest PD and CAL in sampling sites, compared with GCP and C groups. Furthermore, GCP group had significantly decreased concentration of IL-11 than C group, which was in accordance with Yetkin et al. [30] and Johnson et al. [31] results. IL-11 has been shown to inhibit IL-1*β*, TNF-*α*, IL-6, and downregulated LPS-induced cytokines throughout the inhibition of NF-**κ**B expression *in vitro* [32]. Previous investigators demonstrated that IL-11 induced osteoblastic differentiation and bone formation *in vivo* and *in vitro* [33]. It has been reported that the twice-weekly administration of recombinant human IL-11 in periodontal disease model acted by blocking proinflammatory cytokines, leading to a reduction in attachment loss and bone resorption and improvement of the inflammatory reaction [14]. Taken together, the results mentioned above allow us to suggest that IL-11 might be a key mediator in preventing the progressive inflammation leading to periodontal tissue breakdown [30].

In this study the total amount and concentration of IL-11 : IL-17 cytokine ratio was significantly higher in C group compared to both GCP and GAgP groups, and higher in GCP group compared to GAgP, that is, decreased with increasing PD. Johonson et al. [31] reported that IL-11 : IL-17 ratios decreased from 3 mm to >6 mm in diseased gingiva. Moreover, Yetkin et al. [30] showed higher IL-11 : IL-17 ratio in healthy sulcus of C subjects compared to deep pockets of GCP patients suggesting that the anti-inflammatory properties of IL-11 may be impaired in deeper pockets. Yücel et al. [16] also found significantly higher IL-11 : IL-1*β* ratio in gingivitis and C groups compared to GCP group.

Recently, Andrukhov et al. [34] stated that due to differences in the bacterial load different types of periodontal diseases could be associated with specific cytokine profiles. It has been suggested that the plaque bacterial load could determine the type of periodontal disease; that is, *A. actinomycetemcomitans* was associated with GAgP and GCP was associated with the red-complex bacteria [35]. In this study, samples with positive *P. gingivalis* in GAgP and GCP patients showed significantly higher total amounts of IL-17 than negative samples. This agrees with another study where *P. gingivalis* induced a significant increase in IL-17 in periodontitis patients than in gingivitis patients [36]. Considering that IL-17 is capable of inducing the production of TNF-*α* [37], our findings are also supported by Andrukhov et al. [34] who reported that TNF-*α* serum levels were significantly increased in periodontitis patients with high plaque load of *P. gingivalis*.

In conclusion, the current findings suggested a role for IL-17 in the periodontal destruction of GAgP and GCP along with a therapeutic role for IL-11. Further investigations with larger populations are required to clarify the specific contribution of IL-17 to the pathogenesis of periodontitis and to determine the therapeutic benefit of IL-11 for resolution of inflammation in periodontal diseases.

## Figures and Tables

**Table 1:** Sequence of used primers and their PCR product size (bp).

Primers	Sequences	PCR product size (bp)
*A*. *a *	5′-GCTAATACCGCGTAGAGTCGG-3′	443
	5′-ATTTCACACCTCACTTAAAGGT-3′	

*P*. *i *	5′-TTTGTTGGGGAGTAAAGCGGG-3′	575
	5′ TCAACATCTCTGTATCCTGCGT-3′	

*P*. *g *	5′-AGGCAGCTTGCCATACTGCG-3′	404
	5′-ACTGTTAGCAACTACCGATGT-3′	

*T*.* f *	5′-GCGTATGTAACCTGCCCGCA-3′	641
	5′-TGCTTCAGTGTCAGTTATACCT-3′	

*T*. *d *	5′-TAATACCGAATGTGCTCATTTACAT-3′	316
	5′-TCAAAGAAGCATTCCCTCTTCTTCTTA-3′	

**Table 2:** Demographic and clinical characteristics of study groups.

	GCP (*n* = 25)	GAgP (*n* = 25)	C (*n* = 15)	Mann-Whitney *U* test
Mean age, years	40.2 ± 2.65^∗#^	27.53 ± 3.73	25 ± 2.58	<0.001
Gender, M/F	13/12	11/14	7/8	
PI	2.4 ± 0.5^∗#^	1.8 ± 0.9*	0 ± 0	<0.001
GI	2.6 ± 0.5^∗#^	1.7 ± 0.8*	0 ± 0	<0.001
PPD, mm	5.7 ± 0.9*	6 ± 1.4*	1.5 ± 0.5	<0.001
CAL, mm	4.9 ± 0.5^∗#^	7.2 ± 1.1*	0 ± 0	<0.001

Data represented as mean ± SD. PI: plaque index; GI: gingival index; PPD: probing pocket depth; CAL: clinical attachment level.

GCP: generalized chronic periodontitis; GAgP: generalized aggressive periodontitis; C: control.

*Statistically significantly different from the C group.

^#^Statistically significantly different from the GAgP group.

**Table 3:** The gingival crevicular fluid (GCF) total amount (pg) and concentrations (pg/*μ*L) of the cytokines IL-11 and IL-17 (mean ± SD (minimum−maximum)) and GCF volume (*μ*L).

Groups				*P* value from Kruskall-Wallis test
	GCP	GAgP	C
IL-11 (pg)	53.1 ± 20.9 (29–112)	56.5 ± 20.1 (16–80)	49.9 ± 28.8 (14–87)	0.309
IL-11 (pg/*μ*L)	52.1 ± 29.2 (22–115)^∗#^	40.5 ± 19.5 (31–102)^∗#^	106.5 ± 41.5 (56–177)	<0.001
IL-17 (pg)	37.4 ± 8.8 (27–58)^#^	56.4 ± 23.7 (28–104)*	30.5 ± 3.8 (26–38)	<0.001
IL-17 (pg/*μ*L)	57.4 ± 34 (23–164)^∗#^	42.2 ± 10.5 (28–67)^∗#^	84.1 ± 34.9 (42–148)	<0.001
GCF (*μ*L)	1.0 ± 0.2 (0.9–1.3)*	1.3 ± 0.2 (0.5–1.5)*	0.4 ± 0.3 (0.2–0.6)	<0.001

GCP: generalized chronic periodontitis; GAgP: generalized aggressive periodontitis; C: control.

*Statistically significantly different from the C group.

^#^Statistically significantly different from the GAgP group.

**Table 4:** Ratios of the IL-11 : IL-17 in GCF of CP, GAgP, and C subjects.

Cytokine ratio	GCP (*n* = 25)	GAgP (*n* = 25)	C (*n* = 15)	*P* value from Kruskall-Wallis test
IL-11 : IL-17^a^	1.46 : 1*	1.09 : 1*	1.62 : 1	0.011
IL-11 : IL-17^b^	1.01 : 1*	0.99 : 1*	1.30 : 1	0.016

GCP: generalized chronic periodontitis; GAgP: generalized aggressive periodontitis; C: control.

*Statistically significantly different from control group (*P* < 0.05).

^a^Ratio of the total amount of IL-11 to IL-17.

^b^Ratio of the concentration of IL-11 to IL-17.

**Table 5:** The occurrence of different periodontopathic bacteria in the dental plaque of GCP and GAgP groups. Data represent the frequency and percentage of the individuals with given amount of periodontal pathogens in probes of dental plaque.

	GCP (*n* = 25)	GAgP (*n* = 25)	*P* value
*A*. *actinomycetemcomitans *(>10^6^)	8 (32)	11 (44)	<0.001*
*T*. *forsythia *(>10^6^)	19 (76)	18 (72)	>0.05
*T*. *denticola *(>10^6^)	18 (72)	13 (52)	<0.001*
*P*. *gingivalis *(>10^6^)	21 (84)	11 (44)	<0.001*
*P*. *intermedia *(>10^6^)	13 (52)	9 (36)	<0.001*
